# A competing risk survival analysis of the impacts of team formation on goals in professional football

**DOI:** 10.3389/fspor.2024.1323930

**Published:** 2024-06-13

**Authors:** Sebastian Le Coz, Loic Iapteff, Maxime Rioland, Titouan Houde, Christopher Carling, Frank Imbach

**Affiliations:** ^1^ Seenovate, Montpellier, France; ^2^ Université de Lyon, Lyon2, Bron, France; ^3^ Laboratoire Sport, Expertise and Performance INSEP, Paris, France; ^4^ DMeM, Univ Montpellier, INRAe, Montpellier, France

**Keywords:** survival analysis, football, soccer, formations, modeling, game analysis

## Abstract

**Introduction:**

This study investigated the influence of team formation on goal-scoring efficiency through analysing the time required for a goal to be scored in elite football matches.

**Method:**

The analysis was conducted using a comprehensive open access dataset encompassing eight major football competitions, including prestigious events such as the World Cup and the UEFA Champions League. It notably focused on the competing risks framework and employed the Fine and Gray model to account for the interplay between two competing events: team A scoring and team B scoring.

**Results:**

Through analysis of Team A’s goal occurrences, we assessed the offensive capabilities of its formation and the defensive effectiveness of Team B’s composition in relation to the time it took for Team A to score a goal. Findings revealed that teams employing the 4-3-3 and 4-2-3-1 formations outperformed other formations (3-4-3, 3-5-2, 4-4-2, 4-5-1, 5-3-2, 5-4-1) regarding goal-scoring efficiency.

**Discussion:**

By shedding light on the impact of team formation on goal scoring, this research contributes to a deeper understanding of some of the successful strategic aspects of elite football.

## 1 Introduction

Team formation (also refereed to as playing system) plays a crucial role in the outcome of football matches. Player positions within their team’s formation influence the style of play notably with regards to running activity and decision-making processes (1). Previous studies have contributed to understanding team formation and their effects on match outcomes. Hirotsu and Wright (2) conducted a study where a football match was simulated between two teams employing different formations to identify optimal tactical shifts for performance improvement. The authors then compared the results of their simulations with the outcomes of two real matches. Interestingly, the formations used in the actual matches largely deviated from the recommendations provided by the algorithm. However, as the authors rightly pointed out, caution is necessary when drawing conclusive judgments based on just two matches. In another study, Mesoudi (3) focused on the factors influencing managers’ choice of the 4-2-3-1 formation. They observed that managers placed greater emphasis on their recent utilization of a formation, as opposed to considering the formation’s frequency of use across the entire managerial population.

Player positioning within formations has also been extensively studied. Shaw and Glickman (4) developed a formation classification system to identify match formations. Popovych et al. (5) conducted a psycho-diagnostic analysis and discovered that attackers exhibit higher levels of tactical thinking than other players. Recently, some authors investigated the physical and technical performance differences between different tactical formations, highlighting notable disparities among center-backs, full-backs, wide midfielders, central midfielders, and forwards (6).

Furthermore, running performances have been examined within different formations. Research conducted by (7) focused on running performance across formations using three defenders vs. four defenders. Results revealed that formations with three defenders led to higher total and high-intensity running distances for central defenders compared to formations with four defenders.

In evaluating formations and predicting match outcomes, Dobreff et al. (8) used players attribute data and match data from European Soccer to determine the winning probabilities associated with different formations. Their approach focused on home, away, and favored or unfavored teams, using a reward and penalty system to assess formation efficiency. However, their model only used pre-match data and thus did not consider mid-game tactical changes.

In optimizing match outcomes, Bayesian networks were used for modeling the pre-match optimal tactic and a stochastic model for the optimal in-game tactic (9). These algorithms allowed selection of the best formation, players, and playing style, maximizing the team’s chances of winning. Away teams were shown more likely to select tactics that minimize the chances of the opposition winning rather than trying to maximize their chances of winning the game. Even if this model might be adopted by a coach, the influence of a specific formation on goals may be hard to determine as their model lacked interpretability. Consequently, a coach may have difficulties applying the algorithm’s strategy.

If we are to transition from traditional methods to survival analysis, the latter offers a unique perspective in sports statistics by focusing on the time to an event rather than just the event itself. Just as survival analysis aids physicians in assessing death risk and treatment efficacy (10–12), it can provide valuable insights into game dynamics, which traditional statistical methods may overlook, such as the impact of the first goal on subsequent scoring patterns or the time between goals scored or conceded for different teams or formations. Introducing survival analysis into the realm of sports statistics offers a fascinating approach to understanding the dynamics of sports events, such as the time before a team scores a goal.

Survival analysis has already been employed for investigating the effects of the first goal occurrence on the following goal (13). Using a Cox model (14), the authors used several features among the time that passed since the first goal was scored, the time of the first goal in the match, the probability of a home team winning, etc. However, the proportional hazards (PH) assumption was overlooked, and proportionality tests were missing (13). As reported by Fedrizzi et al. (15), survival analysis was also conducted to analyze the number of goals scored in the UEFA EURO 2020 final phase and the time interval between goals. The authors used a Poisson distribution for modeling the number of goals and used the Kaplan-Meier Model (16) to compute the survival curves and the time between goals. Their model focused solely on the overall number of goals scored, without considering which team was responsible for each goal. To estimate the time it takes for a specific team or formation to score a goal, a competitive risk analysis model becomes essential.

The Fine and Gray model (11), one of the references for multivariate analysis of competing risks (17, 18) in survival analysis in biostatistics, was extended to include covariate stratification by Zhou et al. (19) and allows one to examine the offensive capabilities of Team A’s formation and its impact on the time taken for Team A to score a goal. Additionally, one can explore the defensive aspects of Team B’s formation and its effect on the time taken for Team A to score a goal. By incorporating two distinct events, namely, Team A scoring a goal and Team B scoring a goal, one can investigate the intricate dynamics between different formations and their influence on goal-scoring duration. In light of previous studies, our research focuses on examining the influence of team composition on the time required for a goal to be scored. Rather than exploring factors that may affect goal-scoring time, the aim here was to comprehensively understand how team compositions contribute to this aspect. By delving into the dynamics of goal-scoring within different team compositions, we provide valuable insights that can enhance team strategies and overall performance in football.

## 2 Material and methods

### 2.1 Data description

We used the StatsBomb dataset (20) in this study. The data provides information on 753 matches from 8 competitions such as : Champions League (2004, 2005, 2007 and 2009 to 2020), FA Women’s Super League (2018 to 2021), FIFA World Cup (2018 and 2022), Indian Super league (2021/2022), NWSL (2018), UEFA Euro (2021), UEFA Women’s Euro (2022) and Women’s World Cup (2019). The data was collected by five data collectors. One reviewer to check everything is correct, one to collect all the main events, two to tag players and the location of events for each team, and the last person to fill in information about each event. The dataset includes detailed event data such as passes, shots, and red cards.

### 2.2 Pre-processing

First, we extracted from the data the following Key Performance Indicators (KPI) per formation, such as: pressure given, pressure received, mean expected goal per game, variance of the expected goal per game, number of shots taken per game, number of pass per game. The pressure applied, number of shots taken, pressure received, and passes made were determined by extrapolating their values from a match in which the formation wasn’t used for the entire game. Specifically, we computed the mean number of passes P made for a formations as

$$P=\frac{1}{M}\sum_{m}\frac{\;p_{m}}{T_{m}}*T_{T}$$

where M is the number of matchs, $p_{m}$ is the number of passes in the match m, $T_{m}$ is the time playing the formation in the match m and $T_{T}$ is the duration for the match m. We also extracted the same KPI’s for formations against another formation.

The data set preparation followed successive steps. Firstly, each match was segmented into different data points based on events such as halftime, goals, red cards and team formation modifications. The data point was stopped whenever such an event occurred, and a new one was created to continue the match analysis. All events differents from scoring are considered censored data in our analysis. Additionally, considering the absence of a team formation modification following a red card in the StatsBomb dataset, a red card event was treated as equivalent to the end of a match. That helped us to ensure data integrity and avoid anomalies. Moreover, to accommodate the StatsBomb data, which included columns for both “away team” and “home team” even in competitions without designated home teams, we extracted the “Home or away” feature for analysis. We employed data augmentation techniques to expand our dataset and enhance its robustness by doubling our dataset swapping the team columns, ensuring that all corresponding features were adjusted accordingly. This data manipulation allowed us to focus exclusively on the occurrence of “Team A scoring” in the Fine and Gray model. By treating this event as the focal point, we effectively considered the data for a particular formation with Team A scoring as equivalent to data for the same formation with Team B scoring.

The features used in the analysis included: “minutes” at the start of the data point, “period” (representing the period of the match), “Period time” (denoting the starting time in the period of the data point), “Home or away” (with a value of 1 if team A is home, 0 if no team is considered home, and −1 if team B is home), “Goal difference” (indicating the difference in goals), “Gender” (with value of 0 is the game played by men and 1 if played by women), and “Number of Goals” (representing the number of goals scored in the match before the start of the data point). From the team formation provided by StatsBomb, we extracted the number of attackers, midfields and defenders. We also simplified the formations into eight main formations: 3-4-3, 3-5-2, 4-3-3, 4-4-2, 4-5-1, 5-3-2, 5-4-1, 4-2-3-1. The formation 4-2-3-1 was not simplified into a 4-5-1 or a 4-3-3 as it is one of the most played formations as seen in the Supplementary Table S1. As the Fine and Gray model cannot use categorical data, we one-hot encoded all the formations. For replicability, we attached the prepared dataset in the Supplementary Material. We also provided the complete code to recreate the results (21) and the pseudo-code for the pre-processing in Supplementary Material 6.1.

### 2.3 Model definition

The Fine and Gray model (M), is a semi-parametric proportional hazards model that extends the Cox model to estimate the cumulative incidence function (CIF) in the presence of competing risks. Competing risks data occurs when there are multiple possible outcomes, and the occurrence of one outcome precludes the occurrence of the others.

The Fine and Gray model relies on the specific cumulative incidence function and the sub-distribution hazard function. The sub-distribution hazard function calculates the instantaneous risk of event type k given that the individuals at risk are those who have not experienced an event and those who have experienced an event other than event type k. Where as the cumulative incidence function is defined as:

$$I_{k}(t|Z)=P(T\leq t,\Delta =k|Z),\;\forall k\in \{1,\ldots .,K\})$$

where Z is a matrix of covariates, and T is a random variable which corresponds to the time until the occurrence of the first event. The random variable Δ corresponds to the indicator of the type of first event and K is the number of competing events. The sub-distribution hazard function can be interpreted as an instantaneous specific hazard function of the pseudo-random variable $T^{*}$ for:

$$T^{*}=1_{\left\{\Delta =k\right\}}\times T+1_{\left\{\Delta \neq k\right\}}\times \infty$$

The sub-distribution hazard function for event type k is defined by:

$$\gamma_{k}(t|Z)=\lim_{dt\to 0}\frac{1}{dt}P(t\leq T\leq t+dt,\Delta =k|T>t\cup (T\leq t\cap \Delta \neq k\cap C>t),Z)$$

Where C is the random variable corresponds to censoring. The relationship between the sub-distribution hazard function and the cumulative incidence function is:

$$\gamma_{k}(t|Z)=\frac{1}{1-I_{k}(t|Z)}\frac{d(I_{k}(t|Z))}{dt}$$

In the Fine and Gray model, the sub-distribution hazard function specific to event type k corresponds to the product of the baseline sub-distribution function, denoted $\gamma_{k,0}$, and the The sub-distribution hazard function of the event k can be written as follows:

$$\gamma_{k}(t|Z)=\gamma_{k,0}(t)\exp\left(\beta_{k}Z\right)$$

The baseline sub-distribution hazard function corresponds to the sub-distribution hazard function of an individual with a null covariate vector.

The model estimates the effect of covariates on the sub-distribution hazard function, which describes the instantaneous risk of experiencing an event of interest, given that the individual has not yet experienced any event. The sub-distribution hazard ratios obtained from the Fine and Gray model describe the relative effect of covariates on the sub-distribution hazard function. It can also be interpreted as affecting the CIF or the probability of events occurring over time.

The stratification in the Fine and Gray model ($M^{*}$) is achieved by including a stratification variable, denoted as X, in the model. This variable represents the groups or strata based on stratified data. By incorporating the stratification variable, the stratified Fine and Gray model estimates separate baseline sub-distribution hazard functions for each stratum, accounting for potential differences in survival patterns across the strata. The stratification variable does not have associated regression coefficients, but influences the baseline sub-distribution hazard function across the strata.

$$\gamma_{k}(t|Z,X)=\gamma_{k,0}(t|X)\exp\left(\beta_{k}Z\right)$$

where X is a matrix of covariates used in the stratification.

The Fine and Gray model assumes proportional sub-distribution hazard functions. In other words, the relationship between the hazard defined by one set of features over the risk defined by another remains constant over time. Thus, the feature’s impact on the outcome must not change over time. Deviating from the model’s assumptions often leads to biased estimates or incorrect conclusions (22). The advantage of the stratified model is that the covariates X used for stratification are not required to satisfy the proportionality assumption. Stratification ensured that features could still be included even if the model’s proportionality hypotheses were rejected. However, it is essential for the covariates Z to satisfy the proportionality assumption. Since we are only interested in team formation, all the features except team formation were stratified.

In our study, we employed three distinct models to investigate the impact of football formations on the event of interest. The first model, a stratified Fine and Gray model $M^{*}$, was designed to analyze the effect of a single formation at a time. This approach allowed us to precisely assess the influence of each formation on the occurrence of the event. We also used a simple Fine and Gray model M to verify the proportionality hypothesis for the formation and calculate the probability of scoring before a time T. The probability of scoring before a time T has been analysed for goals happening between 10–20 min in the match and between 70–80 min.

In the second model, we incorporated two formations simultaneously, with one formation representing the attacking side and the other serving as the defensive side. This dual-formation model aimed to provide insights into the interplay between different formations during the event.

To further enhance our analysis, the third model introduced a customized feature. This feature, a binary variable, was assigned a value of 1 when both the attack and defense formations were present in a given data point and 0 otherwise.

By utilizing the second and third models, we assessed whether a particular formation exhibited an advantage over another. The objective behind introducing the third model was to address situations where the second model failed to determine the superiority between the attack and defense formations conclusively.

### 2.4 Statistical analysis

In order to select the best-suited model to analyze team formation and avoid over-fitting, we proceeded to a model selection based on the Bayesian Information Criterion (BIC). The BIC is defined as:

BIC=−2ln⁡(L)+kln⁡(n)

where L is the likelihood of the model, n the number of data points, k the number of features. The aim is to select the model that minimizes the BIC. A model selection was applied using the BIC and shown in the results for each formation.

The proportionality assumption of the stratified Fine and Gray model was tested using Li’s test (23). Li’s test is an extension of Lin’s test (24) from a Cox model to a Fine and Gray model. The p-values obtained from the Kolmogorov-Smirnov “prop KS,” Cramer-VonMises “prop CvM,” and the Anderson-Darling “prop AD” tests correspond to Lin’s proportionality tests.

For two groups A and B, these tests compare the sub-distribution hazards for group A with the sub-distribution hazards for group B. In simple terms, for two groups A and B differing by only one covariate, if the distribution of group A is identical to the distribution of group B multiplied by a constant then proportionality is not rejected for the covariate. In other words, these tests assume that the sub-distribution hazards are proportional over time.

If the *p*-value is smaller than a chosen significance level (α), we reject the null hypothesis (H0) and exclude the feature from the analysis. In addition, “*p*-value” is used in the Fine and Gray model to test whether a coefficient equals zero.

The stratified Fine and Gray model estimates the parameter values and tests their significance by comparing them to zero. If the resulting p-value from the Fine and Gray model is below α, we reject the null hypothesis that the parameter equals zero. The significance level of all tests was set at p=0.05 and consistently reported within the analysis.

The hazard ratio (HR) in the context of competing risks analysis can be obtained by exponential coefficients ($e^{\beta_{k}}$) derived from the model. Hence, an exponential hazard ratio greater than 1 indicates an increased hazard, while a hazard ratio less than 1 suggests a decreased hazard. In the case of binary variables representing formations, one can examine the hazard ratio of having a formation compared to not having the formation. This relationship can be expressed as:

$$\frac{\gamma_{k}(t|z=1,X)}{\gamma_{k}(t|z=0,X)}=\frac{\exp\left(\beta_{k}\times 1\right)}{\exp\left(\beta_{k}\times 0\right)}=\exp\left(\beta_{k}\right).$$

## 3 Results

All the features except for team formation were stratified in the model. The model with only the stratified feature “minutes” has the best BIC and has therefor been selected over the other models. An example of the model selection for the 4-3-3 formation is reported in Tables S3, S4 in the Supplementary Material.

The occurrence Table S1 in the Supplementary Material shows that most games were played with the formations 4-2-3-1, 4-3-3 and 4-4-2 and had respectively 2,417, 1,989 and 1,894 games. A closer examination of the data presented in Table 1 reveals a limited number of goals scored or conceded when employing the 5-3-2 and 5-4-1 formations. The 5-4-1 formation scored only seven goals against other formations, and nine goals were conceded. As for the 5-3-2 formation, four goals were conceded, and zero were scored.

**Table 1:** Goals scored by Team A per formation in attack and defense. Event per predictor variable.

Attack	Defense								
	343	352	4231	433	442	451	532	541	Total goals scored
343	16	13	20	23	15	6	0	0	93
352	14	15	36	42	53	22	0	1	183
4231	52	46	185	171	142	91	0	2	689
433	57	52	180	97	146	73	2	3	610
442	21	47	139	103	119	40	2	2	473
451	6	9	50	27	40	19	0	1	152
532	0	0	0	0	0	0	0	0	0
541	0	1	3	3	0	0	0	0	7
Total goals taken	166	183	613	466	515	251	4	9	

The 4-5-1 formation also reported a limited number of goals scored or conceded against specific formations. The formation 4-5-1 only scored six and nine goals against the 3-4-3 and 3-5-2 formations respectively while it only six were conceded against a 3-4-3. The formation 4-2-3-1 recorded the most goals scored and conceded. The occurrence of goal scored and conceded for each formation showed significant heterogeneity, as presented in Table 1. The lack of events raises concerns regarding the reliability of the estimations derived from such cases. According to research by Austin et al. (25), it is mandatory to have a minimum of 10 events per predictor variable (EPV) to ensure precise estimations. Given the insufficient number of events associated with the 5-3-2 and 5-4-1 formations, estimates should be interpreted with caution.

The KPI noticed in the Table 2 show that the mean expected goal for any formation is around 10% and the variance of the expected goal is around 2%. The number of passes vary between 303 and 504 passes and the number of shots varies between 6.5 and 11.5 shots. The formations 4-3-3, 4-2-3-1 followed by the 4-4-2 have the most passes, shots taken and pressure received. The 4-3-3 always outperforms the other compositions in these 3 KPI. The formation with the most pressure given is the 4-4-2 and seems to be the best defensive formation as it has the highest pressure given. However, the formation negating the most shots from the enemy team is the 4-3-3. The main difference between the 4-4-2 and the 4-3-3 seems to reside in the pressure placed on other formations. The 4-2-3-1 has one of the highest pressure placed to other formations whereas the 4-3-3 has one of the lowest. The formations 4-3-3 and 4-2-3-1 show a pass-based game-play as they are often pressured by other formations and takes numerous shots.

**Table 2:** KPI representation for each formation.

Formation	Pressure received	Pressure given	Mean xg	Variance xg	Pass	Shots
451	148.672	161.576	0.101	0.021	401.228	9.151
343	139.336	161.199	0.102	0.022	395.987	8.859
433	**175.158**	156.486	0.103	0.023	504.751	11.470
442	169.216	175.044	0.102	0.023	459.218	11.222
4231	**176.298**	169.712	0.105	0.023	485.611	11.301
352	159.960	164.537	0.104	0.025	442.660	10.337
541	132.637	153.561	0.108	0.018	337.085	7.071
532	150.558	170.362	0.102	0.019	303.059	6.663

The results presented in Table 3 indicate that the formations Attack 5-3-2 and Attack 3-5-2 do not exhibit proportionality and should be excluded from consideration. Additionally, formations such as Defense 3-5-2, Attack 3-5-2, Defense 4-4-2, Attack 4-4-2, Defense 5-3-2, Defense 5-4-1, Attack 4-2-3-1 and Attack 5-4-1 have limited impact on the model (p>0.05). The formations in bold in the Table 3 Defense 4-3-3, Attack 4-3-3, and Defense 4-2-3-1 are the only favorable formations (p<0.01, HR=0.81∈[0.73,0.90],:95%CI; (*p* = 0.01, HR = 1.25 ∈ [1.14, 1.37],:95%CI; (p<0.03, HR=0.9∈[0.82,0.98],:95%CI for Defense 4-3-3, Attack 4-3-3, and Defense 4-2-3-1, respectively). The hazard ratio of the Defense formation in the 4-3-3 strategy is smaller than 1, indicating that this formation decreases the likelihood of the event (e.g., the attacking team scoring). Conversely, the HR of the Attack formation in the 4-3-3 strategy is greater than 1, implying that this formation increased the likelihood of the team scoring. The Defense formation 4-2-3-1 shows an HR smaller than 1. It suggests that this formation exposes a qualitative defense. The defense formations 3-4-3 and 4-5-1 exhibit HR values greater than one. That indicates these formations are defensively inefficient. In addition, 3-4-3 and 4-5-1 offensive formations showed low HR values, suggesting weak offensive skills. Given large p values, the Defense 3-5-2, 4-4-2 and Attack 4-2-3-1 and 4-4-2 have limited impact on the model. Therefore, we cannot state whether these formations are efficient or ineffective.

**Table 3:** Fine and gray estimation for each formation.

Formation	prop KS	prop CvM	prop AD	BIC	*p*-value	HR	CI
Defense 343	0.44	0.49	0.47	38,864.80	0.00	1.34	[1.15, 1.57]
Defense 352	0.69	0.75	0.87	38,876.74	0.99	1.00	[0.86, 1.16]
Defense 4231	**0.95**	**0.99**	**0.99**	**38,871.37**	**0.02**	**0.90**	**[0.82, 0.98]**
Defense 433	**0.38**	**0.36**	**0.56**	**38,860.51**	**0.00**	**0.81**	**[0.73, 0.90]**
Defense 442	0.52	0.46	0.42	38,874.64	0.14	1.08	[0.98, 1.19]
Defense 451	0.12	0.11	0.12	38,860.11	0.00	1.33	[1.17, 1.51]
Defense 532	0.40	0.32	0.31	38,875.88	0.25	1.66	[0.69, 3.97]
Defense 541	0.12	0.14	0.15	38,874.04	0.08	1.84	[0.92, 3.68]
Attack 343	0.68	0.55	0.54	38,856.40	0.00	0.64	[0.52, 0.78]
Attack 352	0.00	0.00	0.00	38,876.59	0.70	1.03	[0.88, 1.20]
Attack 4231	0.90	0.92	0.77	38,873.49	0.06	1.09	[0.99, 1.37]
Attack 433	**0.70**	**0.71**	**0.73**	**38,855.56**	**0.00**	**1.25**	**[1.14, 1.37]**
Attack 442	0.44	0.22	0.32	38,875.41	0.24	0.94	[0.85, 1.04]
Attack 451	0.22	0.22	0.21	38,854.10	0.00	0.68	[0.58, 0.80]
Attack 532	0.00	0.00	0.00	38,869.98	0.00	0.00	[0.00, 0.00]
Attack 541	0.15	0.22	0.27	38,876.66	0.76	1.12	[0.55, 2.27]

Favorable formations are displayed in bold. The Kolmogorov-Smirnov test is noted as “prop KS.” The Cramer-VonMises test is noted as “prop CvM” and the Anderson-Darling test is noted as “prop AD.” “HZ” Corresponds to the Hazard Ratio and “CI” to the confidence interval.

**Figure 1:**
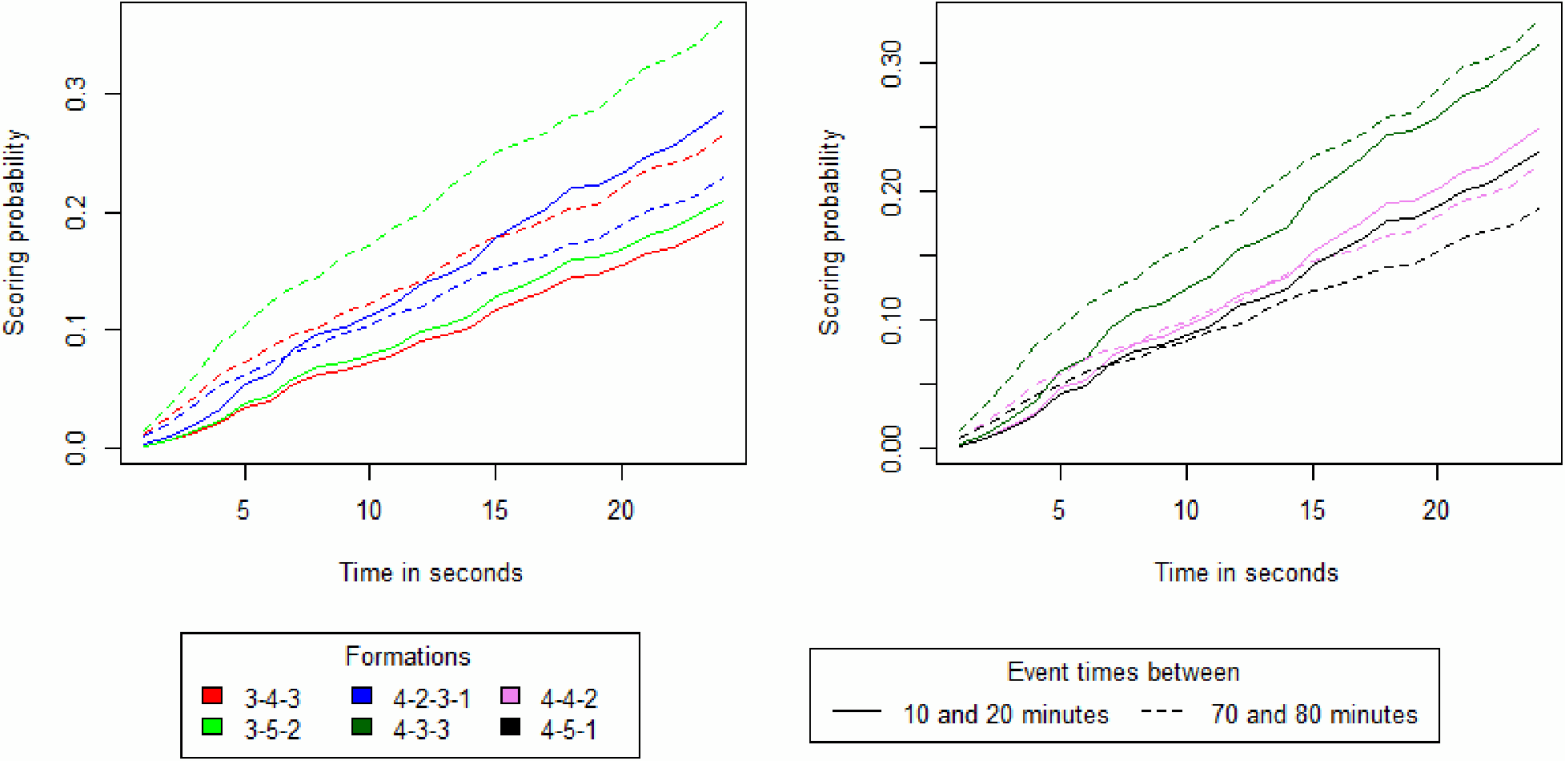
Survival curves depicting the probability of scoring a goal over time, segmented by team compositions and match time frames.

The Figure 1 is the cumulative distribution function which represents the probability of scoring before a time T under the hypothesis that no other event happens than the team in possession scoring. The probability of scoring a goal over time is segmented by team compositions and for events between 10 and 20 min and 70 and 80  min. The 5-4-1 and 5-3-2 formations have been excluded from this figure as they did not report enough scored goals during these periods. The results show that at the start of a data point the probability of scoring is higher in later stages of a match than in the beginning. For the team formation 4-2-3-1, 4-3-3 and 4-5-1 the trend inverts if the data point is not interrupted by an event after 15 s.

Table 4 presents models 2, 3, highlighting the potential outcome of two distinct formations competing against one another. First, the third model demonstrated superior performance based on the BIC compared to the second model. However, the third model would often lead to inconclusive results when the second model was conclusive. For instance, considering the Attack 4-4-2 and Defense 3-4-3, the *p*-values reported in the model 3, 2 suggest a feature withdraw for Attack 4-4-2 (p=0.31 and p=0.54), while the Defense 3-4-3 should be considered (p<0.01). In this case, the results of the formation “Defense 3-4-3” will entirely determine the outcome. Details of the main formation couples are displayed within Table 4.

**Table 4:** Table of formations competing against one another.

Formation		Model 2						Model 3		
		Attack			Defense			Attack and defense		
Att	Def	*p*-value	HR	CI	*p*-value	HR	CI	*p*-value	HR	CI
343	343	0.00	0.63	[0.51,0.77]	0.00	1.37	[1.17,1.6]	0.91	0.97	[0.6,1.56]
**4231**	343	0.05	1.09	[1,1.2]	0.00	1.35	[1.15,1.57]	0.00	1.74	[1.32,2.28]
**433**	343	0.00	1.24	[1.13,1.36]	0.00	1.32	[1.13,1.54]	0.00	1.54	[1.2,1.99]
**442**	343	0.31	0.95	[0.86,1.05]	0.00	1.34	[1.14,1.56]	0.54	1.14	[0.75,1.73]
451	343	0.00	0.69	[0.58,0.81]	0.00	1.32	[1.13,1.55]	0.55	0.79	[0.36,1.73]
343	**352**	0.00	0.64	[0.52,0.78]	0.91	1.01	[0.87,1.17]	0.48	0.83	[0.49,1.4]
**433**	352	0.00	1.25	[1.14,1.37]	0.97	1.00	[0.86,1.16]	0.18	1.20	[0.92,1.56]
343	**4231**	0.00	0.64	[0.52,0.78]	0.02	0.89	[0.81,0.98]	0.00	0.50	[0.33,0.77]
4231	**4231**	0.08	1.08	[0.99,1.19]	0.02	0.90	[0.82,0.98]	0.88	0.99	[0.85,1.15]
433	4231	0.00	1.26	[1.15,1.38]	0.01	0.89	[0.81,0.97]	0.61	1.04	[0.89,1.21]
442	**4231**	0.25	0.94	[0.85,1.04]	0.02	0.90	[0.82,0.98]	0.43	0.94	[0.79,1.1]
451	**4231**	0.00	0.69	[0.58,0.81]	0.03	0.90	[0.82,0.99]	0.02	0.71	[0.54,0.94]
343	**433**	0.00	0.65	[0.53,0.79]	0.00	0.82	[0.74,0.91]	0.00	0.53	[0.35,0.8]
433	433	0.00	1.23	[1.12,1.35]	0.00	0.83	[0.75,0.92]	0.71	1.04	[0.85,1.28]
4231	433	0.04	1.10	[1,1.2]	0.00	0.81	[0.73,0.9]	0.57	0.96	[0.82,1.12]
442	**433**	0.28	0.95	[0.86,1.05]	0.00	0.81	[0.74,0.9]	0.02	0.79	[0.65,0.96]
451	**433**	0.00	0.68	[0.58,0.8]	0.00	0.81	[0.74,0.9]	0.00	0.49	[0.34,0.72]
343	**442**	0.00	0.64	[0.52,0.79]	0.21	1.06	[0.97,1.17]	0.30	0.77	[0.46,1.27]
**433**	442	0.00	1.25	[1.14,1.37]	0.17	1.07	[0.97,1.18]	0.02	1.22	[1.04,1.44]
451	**442**	0.00	0.68	[0.58,0.8]	0.16	1.07	[0.97,1.18]	0.62	0.92	[0.68,1.26]
343	451	0.00	0.64	[0.53,0.79]	0.00	1.32	[1.16,1.5]	0.52	0.77	[0.34,1.73]
**4231**	451	0.08	1.08	[0.99,1.18]	0.00	1.33	[1.17,1.51]	0.00	1.44	[1.18,1.77]
**433**	451	0.00	1.25	[1.14,1.37]	0.00	1.33	[1.17,1.52]	0.00	1.68	[1.34,2.11]
**442**	451	0.25	0.94	[0.85,1.04]	0.00	1.33	[1.17,1.51]	0.69	0.94	[0.69,1.28]
451	451	0.00	0.68	[0.58,0.8]	0.00	1.33	[1.17,1.52]	0.35	0.81	[0.53,1.25]

Winning formations are displayed in bold, whereas regular couples do not permit a clear identification of the winners.

In Table 4 and due to considerations of proportionality, *p* values, and the requirement for a sufficient number of EPV, some formation couples (attack and defense) had to be disregarded. The formations in bold in Table 4 have an edge over the formations they are competing against. The results show that the formation 4-3-3 consistently outperforms other formations except against the 4-2-3-1 and itself. A similar observation is noticed for the 4-2-3-1. However, the defense formation 4-2-3-1 outperforms the attack 4-2-3-1. The results show that formation 4-4-2 is efficient against formations 3-4-3 and 4-5-1. The last formation that stands out is the 3-5-2. It only wins in defense situations against the 3-4-3.

## 4 Discussion

In this study, we employed a competitive risk model, a previously unexplored approach in prior research, to provide insights into how team formation affect goal-scoring time and highlight advantageous formations within a competitive context. Using the Fine and Gray competitive risk model with stratification ensures a robust methodology to assess a formation’s influence on the duration required for a goal to be scored.

The formation analysis conducted by Dobreff et al. (8) did not encompass the 4-2-3-1 formation, yet yielded comparable outcomes for the 4-3-3 formation, emerging as one of the most effective formations to win a game. However, in contrast to our results, Dobreff et al. (8) found the 4-4-2 formation to be mainly effective when playing at home against the 4-5-1 formation, and vice versa, while the 4-5-1 formation demonstrated its effectiveness exclusively at home against the 4-4-2 formation.

Our results have shown that the best formations are 4-3-3 and 4-3-2-1. They both have high number of passes, shots taken and pressure received compared to other formations. However, they differ on the pressure placed on opponents. Our analysis highlights 4-3-3 as an offensive-like formation where 4-3-2-1 is more defensive. Additionally, our results suggest that the best defense is a good offense.

Since the dataset used in this study (20) encompassed a rich collection of actions that transpired during a football game (including passes, shots, and various types of shots), we tried to widen our event space by including possession changes and shots taken to conduct a deeper analysis of the impact of the formations on these new events. However, we obtained similar results where the 4-3-3 formation was dominant at intercepting the ball, taking shots and limiting the shots the enemy team took.

Previous research by Shaw and Glickman (4) demonstrates that teams possessing the ball tend to spread out more than teams in a defensive posture. Additionally, the playing style and tactics will vary with the state of the ongoing game, as modeled by (9) and analysed by (26). Switching a teams composition or stategie between two events is not taken into acount in our model. Hence, a variation in play style could have been the cause for the attacking 3-5-2 formation to not satisfy the proportionality test whereas this was the case for the other formations.

The playing style of a team is not static and may evolve not only between events but also during the course of a game, often due to time pressure in an attempt to maintain an advantage or make a comeback, as suggested by Ric et al. (27). This time pressure becomes particularly critical in the last 15 minutes of football matches, a phase of the game as being more likely to produce goals as shown in our results and identified by Simiyu (28). Additionally, teams that are either evenly matched or trailing in a game may experience improved performance for the subsequent 10 min if they modify their formation, according to research by Forcher et al. (29).

In an effort to account for the impact of time pressure, the starting time of the data point has been incorporated into the model via stratification. However, this stratification presents a challenge as it disables the potential analysis of the impact of the starting time on the effect of formations on goal scoring times. Furthermore, one could argue that the advantage of formation modification described by Forcher et al. (29) is not fully considered when stratifying the starting time. This suggests that while time pressure is a crucial factor in the evolution of a team’s playing style, the complex interplay between time, formation changes, and goal scoring requires further investigation.

A number of championships such as “La Liga” were discared from the study as the dataset only included the matchs played by the best team. If used these could have created a biase as the team formation would have been disproportionately represented as few formations were consistently played by the best team. However no analysis has been done to measure impact of the top 5 teams on our results. In order evaluate the impact of team strength one could create a ranking base on the sum of the players ranking and analyse the correlation between the ranking and the team composition.

To extend the research scope, it is worth considering alternative events, decomposing the goal event, and incorporating supplementary events such as the occurrence of red and yellow cards to deepen the analysis. Additionally, exploring different features characterizing players or teams and their influence on goal-scoring time, presents another promising avenue for future exploration. Expanding the scope of investigation not only enhances the richness and granularity of the analysis but also presents opportunities for a more nuanced understanding of the intricate factors that shape the outcomes of football matches. By including additional events future studies can contribute to developing strategies, tactics, and formations that optimize goal-scoring capabilities, inform team selection, and potentially uncover hidden patterns within the intricate fabric of football dynamics.

## 5 Conclusion

Based on our survival analysis model, the Fine and Gray model was employed to determine goal times, revealing that the 4-3-3 and the 4-2-3-1 formation in attack and both the 4-3-3 and 4-2-3-1 formations in defense showcased remarkable success rates, surpassing other formations significantly. However, no definitive distinction could be made between these two formations as the overall “winner.” Notably, teams adopting the 4-3-3 and 4-2-3-1 formations exhibited exceptional defensive capabilities, rendering goal-scoring against them exceptionally challenging. The 4-2-3-1 formation demonstrated exceptional defensive capabilities against teams employing the same formation. Furthermore, the 4-3-3 formation displayed notable offensive potential, providing teams with optimal opportunities to score goals.

In summary, our study highlights the significance of team composition in achieving favorable goal outcomes in football matches, underscoring the supremacy of the 4-3-3 formation and its potential as a strategic blueprint for enhancing goal-scoring potential. While the significance of playing formation is acknowledged throughout the world of football, the innovative nature of our study lies in its application of the Fine and Gray model to extract relevant information from matches. This shift from traditional analyses by using competing risk survival analysis promises to revolutionize our understanding of how teams shape the outcome of a match. Accordingly, understanding the impact of team compositions on the duration required for a goal to be scored is essential for optimizing match outcomes and improving overall performance.

## Data Availability

The original contributions presented in the study are included in the article/**Supplementary Material**, further inquiries can be directed to the corresponding authors.
