# Supplementary material for: A competing risk survival analysis of the impacts of team formation on goals in professional football
Source: Front Sports Act Living. 2024 Jun 13;6:1323930. doi: 10.3389/fspor.2024.1323930 (PMC11208451; doi:10.3389/fspor.2024.1323930)
Supplement: Supplementary file 2 [file Presentation2.zip › sup.pdf]

1 SUPPLEMENTARY DATA

1.1 Pre-processing pseudo-code

```
New data = empty matrix
for Competition in Data do
    for Match in Competition do
        for Event in Match do
            Filter Events to keep lines with red cards, last event in period, formation changes, and goals.
            Delete Events that occur after red cards.
            Create EventFineandGray with red cards, last event in period, formation changes, and goals.
            Create TimeFineandGray with Event.
            Create Feature Minutes with Event.
            Get Feature FormationteamA from Event.
            Get Feature FormationteamB from Event.
            Modify Features FormationteamB and FormationteamA using a correspondence table.
            One hot encode Features FormationteamB and FormationteamA
            Get Feature period from Event.
            Create Feature Home or away from Event, Competition, and Match.
            Create Feature Number of Goals from Event.
            Create Feature Goal difference from Event.
            Perform Data Augmentation by inverting FormationteamA and FormationteamB.
            Modify Home or away, Goal difference, and EventFineandGray accordingly.
            data = All Features and Fine and gray features
            New data = New data bind rows with data
        end for
    end for
end for
Result = Fine and Gray(OnehotFormation)
check Proportionality of Result
```

1.2 Tables

Formation	343	352	4231	433	442	451	532	541
343	68	57	137	155	82	27	0	1
352	57	74	169	177	181	62	0	5
4231	137	169	672	643	531	257	0	8
433	155	177	643	342	473	184	5	10
442	82	181	531	473	460	159	5	3
451	27	62	257	184	159	78	1	2
532	0	0	0	5	5	1	0	0
541	1	5	8	10	3	2	0	0
Total	527	725	2417	1989	1894	770	11	29

Table 1. The Occurrence table of each formation

F_i	Press_rec_i	xg_i	var_xg_i	pass_i	shots_i	F_j	Press_rec_j	xg_j	var_xg_j	pass_j	shots_j
451	152.22	0.10	0.02	423.39	9.56	451	152.22	0.10	0.02	423.39	9.56
451	164.37	0.10	0.02	520.88	9.68	343	140.07	0.09	0.02	396.29	6.55
451	132.26	0.10	0.02	342.64	7.62	433	160.40	0.09	0.02	526.09	10.17
451	144.10	0.10	0.02	395.08	9.68	442	149.35	0.11	0.02	438.48	9.98
451	140.69	0.09	0.02	362.85	8.77	4231	158.63	0.11	0.02	461.27	10.56
451	155.28	0.10	0.02	439.49	12.45	352	147.96	0.09	0.02	418.82	10.08
451	77.16	0.10	0.02	161.33	0.00	541	140.29	0.09	0.02	315.65	14.03
451	190.35	0.10	0.02	403.08	33.59	532	190.35	0.09	0.02	347.10	0.00
343	139.97	0.11	0.02	452.61	8.85	343	139.97	0.11	0.02	452.61	8.85
343	123.64	0.10	0.02	363.61	7.50	433	154.74	0.10	0.02	518.04	11.64
343	134.69	0.12	0.03	383.37	9.51	442	146.46	0.11	0.02	428.10	12.01
343	125.57	0.11	0.03	325.25	7.42	4231	159.56	0.11	0.02	494.94	12.49
343	143.31	0.11	0.03	416.43	8.95	352	155.77	0.10	0.02	459.66	11.04
343	295.21	0.12	0.03	423.56	38.51	541	115.52	0.10	0.02	128.35	0.00
433	156.01	0.10	0.02	434.18	9.67	433	156.01	0.10	0.02	434.18	9.67
433	156.95	0.10	0.02	449.71	11.32	442	140.62	0.10	0.02	373.02	9.19
433	158.40	0.11	0.02	436.46	9.94	4231	148.19	0.10	0.02	398.39	9.11
433	158.26	0.10	0.02	463.39	10.22	352	151.48	0.09	0.02	393.43	8.57
433	220.46	0.10	0.02	367.94	6.61	541	64.91	0.10	0.02	273.26	3.67
433	208.23	0.10	0.02	360.25	17.88	532	124.83	0.10	0.02	309.42	7.80
442	148.75	0.10	0.02	398.52	10.00	442	148.75	0.10	0.02	398.52	10.00
442	143.88	0.10	0.02	380.08	9.33	4231	155.55	0.11	0.02	422.42	9.89
442	148.19	0.10	0.03	418.42	10.22	352	139.12	0.10	0.03	389.87	9.83
442	199.41	0.10	0.02	662.82	16.85	541	109.53	0.10	0.02	219.07	2.81
442	133.54	0.10	0.02	363.80	13.58	532	177.81	0.10	0.02	297.56	5.77
4231	154.21	0.10	0.02	397.88	9.53	4231	154.21	0.10	0.02	397.88	9.53
4231	147.42	0.10	0.02	445.35	10.68	352	141.32	0.10	0.02	384.67	9.26
4231	54.50	0.10	0.02	340.11	5.50	541	213.11	0.10	0.02	549.23	10.00
352	151.99	0.10	0.02	419.96	8.90	352	151.99	0.10	0.02	419.96	8.90
352	239.83	0.09	0.02	470.13	18.99	541	136.05	0.11	0.03	229.82	7.74

Table 2. The KPI table per formation

Features	BIC
**Defense_433_minute**	**38860.51**
Defense_433_period	51070.09
Defense_433_Gender	51346.81
Defense_433_number_of_goals_before	47000.39
Defense_433_Goal_diff	45301.67
Defense_433_time_period	40078.87
Defense_433_Full_Home_away	49471.85
Defense_433_minute_period	46841.48
Defense_433_minute_Gender	44369.10
Defense_433_minute_number_of_goals_before	42707.04
Defense_433_minute_Goal_diff	39047.43
Defense_433_minute_time_period	45082.79
Defense_433_minute_Full_Home_away	42491.12
Defense_433_period_number_of_goals_before	52781.08
Defense_433_period_Goal_diff	50413.81
Defense_433_period_time_period	46777.85
Defense_433_period_Full_Home_away	54615.47
Defense_433_number_of_goals_before_Goal_diff	47370.01
Defense_433_number_of_goals_before_time_period	43453.81
Defense_433_number_of_goals_before_Full_Home_away	50538.37
Defense_433_Goal_diff_time_period	40109.05
Defense_433_Goal_diff_Full_Home_away	48719.07
Defense_433_time_period_Full_Home_away	43670.69
Defense_433_minute_period_number_of_goals_before	50749.38
Defense_433_minute_period_Goal_diff	47104.87
Defense_433_minute_period_time_period	53071.44
Defense_433_minute_period_Full_Home_away	50499.36
Defense_433_minute_number_of_goals_before_Goal_diff	44433.13
Defense_433_minute_number_of_goals_before_time_period	49683.78
Defense_433_minute_number_of_goals_before_Full_Home_away	46631.99

Table 3. Results for the 4-3-3 formation. Model selection with the BIC.

Features	BIC
Defense_433_minute_Goal_diff_time_period	45895.78
Defense_433_minute_Goal_diff_Full_Home_away	43514.21
Defense_433_minute_time_period_Full_Home_away	48899.10
Defense_433_period_number_of_goals_before_Goal_diff	53259.54
Defense_433_period_number_of_goals_before_time_period	50665.55
Defense_433_period_number_of_goals_before_Full_Home_away	56357.67
Defense_433_period_Goal_diff_time_period	46988.24
Defense_433_period_Goal_diff_Full_Home_away	53990.71
Defense_433_period_time_period_Full_Home_away	50417.98
Defense_433_number_of_goals_before_Goal_diff_time_period	44979.95
Defense_433_number_of_goals_before_Goal_diff_Full_Home_away	51877.18
Defense_433_number_of_goals_before_time_period_Full_Home_away	47331.98
Defense_433_Goal_diff_time_period_Full_Home_away	44327.19
Defense_433_minute_period_number_of_goals_before_Goal_diff	52539.38
Defense_433_minute_period_number_of_goals_before_time_period	57725.81
Defense_433_minute_period_number_of_goals_before_Full_Home_away	54705.71
Defense_433_minute_period_Goal_diff_time_period	53955.26
Defense_433_minute_period_Goal_diff_Full_Home_away	51641.42
Defense_433_minute_period_time_period_Full_Home_away	56915.76
Defense_433_minute_number_of_goals_before_Goal_diff_time_period	52032.18
Defense_433_minute_number_of_goals_before_Goal_diff_Full_Home_away	50039.04
Defense_433_minute_number_of_goals_before_time_period_Full_Home_away	53919.23
Defense_433_minute_Goal_diff_time_period_Full_Home_away	50890.37
Defense_433_period_number_of_goals_before_Goal_diff_time_period	52464.95
Defense_433_period_number_of_goals_before_Goal_diff_Full_Home_away	57833.46
Defense_433_period_number_of_goals_before_time_period_Full_Home_away	54628.74
Defense_433_period_Goal_diff_time_period_Full_Home_away	51527.82
Defense_433_number_of_goals_before_Goal_diff_time_period_Full_Home_away	50448.59
Defense_433_minute_period_number_of_goals_before_Goal_diff_time_period	60137.76
Defense_433_minute_period_number_of_goals_before_Goal_diff_Full_Home_away	58193.19
Defense_433_minute_period_number_of_goals_before_time_period_Full_Home_away	61993.56
Defense_433_minute_period_Goal_diff_time_period_Full_Home_away	59017.70
Defense_433_minute_number_of_goals_before_Goal_diff_time_period_Full_Home_away	57929.29
Defense_433_period_number_of_goals_before_Goal_diff_time_period_Full_Home_away	58131.81
Defense_433_minute_period_number_of_goals_before_Goal_diff_time_period_Full_Home_away	66083.53

Table 4. Results for the 4-3-3 formation. Model selection with the BIC.
